# Evolutionary History of Plant LysM Receptor Proteins Related to Root Endosymbiosis

**DOI:** 10.3389/fpls.2018.00923

**Published:** 2018-07-04

**Authors:** Clare Gough, Ludovic Cottret, Benoit Lefebvre, Jean-Jacques Bono

**Affiliations:** LIPM, Université de Toulouse, INRA, CNRS, Castanet-Tolosan, France

**Keywords:** LysM domain, kinase domain, NFP, LYR3, LYK3, nodulation, arbuscular mycorrhization, lipo-chitooligosaccharide

## Abstract

LysM receptor-like kinases (LysM-RLKs), which are specific to plants, can control establishment of both the arbuscular mycorrhizal (AM) and the rhizobium-legume (RL) symbioses in response to signal molecules produced, respectively, by the fungal and bacterial symbiotic partners. While most studies on these proteins have been performed in legume species, there are also important findings that demonstrate the roles of LysM-RLKs in controlling symbiosis in non-legume plants. Phylogenomic studies, which have revealed the presence or absence of certain LysM-RLKs among different plant species, have provided insight into the evolutionary mechanisms underlying both the acquisition and the loss of symbiotic properties. The role of a key nodulation LysM-RLK, NFP/NFR5, in legume plants has thus probably been co-opted from an ancestral role in the AM symbiosis, and has been lost in most plant species that have lost the ability to establish the AM or the RL symbiosis. Another LysM-RLK, LYK3/NFR1, that controls the RL symbiosis probably became neo-functionalised following two rounds of gene duplication. Evidence suggests that a third LysM-RLK, LYR3/LYS12, is also implicated in perceiving microbial symbiotic signals, and this protein could have roles in symbiosis and/or plant immunity in different plant species. By focusing on these three LysM-RLKs that are widespread in plants we review their evolutionary history and what this can tell us about the evolution of both the RL and the AM symbioses.

## Introduction

In plants, several LysM receptor-like kinases (LysM-RLKs) have been characterised as symbiotic receptor proteins. After an introduction about the evolutionary origins of LysM-RLKs, we focus on three LysM-RLKs, known as NFP/NFR5, LYK3/NFR1, and LYR3/LYS12 in *Medicago truncatula*/*Lotus japonicus*, and their hypothetical evolutionary histories related to root endosymbiosis establishment. Other LysM-RLKs described in the text are listed in the glossary.

## Evolutionary Origins of LysM-RLKs

**FIGURE 1:**
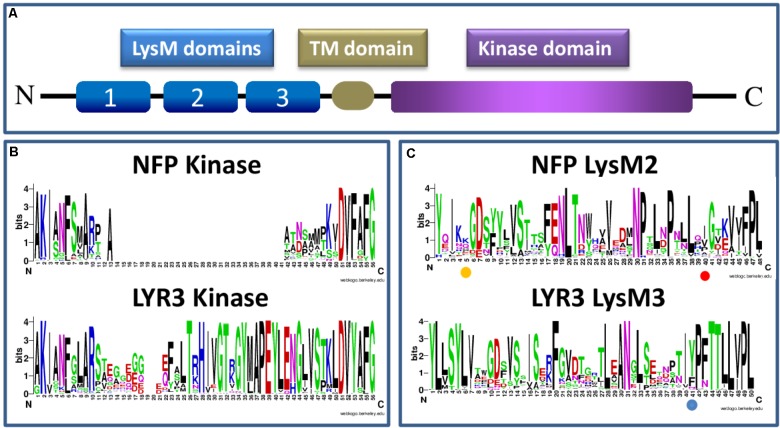
Schematic presentation of a LysM-RLK protein structure **(A)** and sequence logos generated using Clustal W (Larkin et al., 2007) and WebLogo software (Schneider and Stephens, 1990; Crooks et al., 2004), and approximately 60 Angiosperm protein sequences each of NFP or LYR3 **(B,C)** (**Supplementary Table S1** and **Supplementary Data Sheet S1**). The sequence logos show the extent of sequence conservation within **(B)** part of the intracellular domain that contains the activation loop in LYR3 proteins but not in NFP proteins; and **(C)** individual LysM domains (LysM2 for NFP proteins and LysM3 for LYR3 proteins). In **(B)** the conserved “F” at position 6 of the sequence logos corresponds to the “F” in the “DFG” motif of active kinases. The amino acid positions highlighted in **(C)** correspond to L118 in LjNFR5 LysM2; L154 in MtNFP LysM2 and Y228 in MtLYR3 LysM3, as discussed in the text. The LysM sequence logos also show that each LysM domain is a combination of highly conserved and highly variable residues, many of which are probably involved in tertiary structure and ligand binding specificity, respectively. Sequence logos of all the 3 LysM domains of NFP and LYR3 are shown in **Supplementary Figures S1**, **S2**.

Proteins incorporating three extracellular LysM domains, a transmembrane domain and an intracellular kinase domain (**Figure 1A**) are specific to plants, and result from evolutionary events that apparently predate plant colonization of the land (Delaux et al., 2015). Whether the LysM triplet was only formed once is difficult to know, but LysM1, LysM2, and LysM3 of each proteinusually resemble more the same domain of homologous LysM-RLK proteins than other domains of the same protein (Arrighi et al., 2006). LysM domains are of bacterial origin, but unlike prokaryotic proteins with repeated LysM domains, those of LysM-RLKs are always separated by conserved cysteine-X-cysteine motifs, responsible for the formation of disulphide bridges (Lefebvre et al., 2012).

Three types of kinase domain can be distinguished for LysM-RLKs, two of which are predicted to have kinase activity, while a third group can be considered to be pseudo-kinases (Arrighi et al., 2006). These all have a common evolutionary origin, since all kinase domains of plant RLKs form a monophyletic family within the superfamily of plant kinases (Shiu and Bleecker, 2001). Symbiotic LysM-RLKs have so far been identified as “LYK-type” (active kinase) or “LYR-type” (pseudo-kinase) proteins. The presence of both types in all higher plants, together with strong conservation of intron-exon structure, indicates their ancient evolutionary origin and ancient events of duplication. Canonical 3-D structures can be predicted for LYR-type kinase domains (Arrighi et al., 2006), indicating that they evolved from active kinase domains. From our sequence analyses, the first changes were probably loss of the glycine-rich loop and the “DFG” motif at the start of the activation loop, which are ubiquitously absent in such proteins. Subsequently, one evolutionary line leads to proteins such as NFP that have lost the activation loop in their kinase domains, and another leads to most other LYR-type proteins, which have a conserved activation loop. The loss of the activation loop in NFP is common to all plant species analysed here, as shown in the sequence logos in **Figure 1B**, which include the basal flowering plant *Amborella trichopoda* estimated to have split from other flowering plants 145-210 MYA(Aoki et al., 2004). These sequence logos also show good sequence conservation in the activation loop region among all the LYR3 proteins analysed (**Figure 1B**).

## The Evolutionary Acquisition and Loss of Symbiotic NFP Proteins

NFP proteins have been shown to play important roles in establishment of the arbuscular mycorrhizal (AM) symbiosis in the non-legume dicot plants *Parasponia andersonni* (Op den Camp et al., 2010) and *Solanum lycopersicum* (Buendia et al., 2016), and they control nodulation in model legume plants (Gough and Cullimore, 2011). There is a lot of evidence that NFP proteins first controlled the AM symbiosis that evolved approximately 400 MYA, and were subsequently recruited by legume plants so that bacteria could activate symbiotic signalling pathways that were adapted to allow nodulation (Geurts et al., 2016), which appeared approximately 65 MYA. The presence of a pseudo-kinase in a LysM-RLK (LYR-type) involved in both symbioses suggests signal transduction occurs via protein/protein interactions. At least in the model legumes *M. truncatula* and *L. japonicus*, it is likely that heterodimerisation of NFP/NFR5 proteins with the kinase-active LYK3/NFR1 LysM-RLK forms a receptor complex (Madsen et al., 2011; Pietraszewska-Bogiel et al., 2013; Moling et al., 2014).

Legume NFP proteins are essential for the perception of rhizobial Nod factors, which are lipo-chitooligosaccharide (LCO) signal molecules, consisting of an *N*-acetyl glucosamine backbone and an acyl chain. In acquiring the ability to synthesise LCOs, rhizobial bacteria mimicked the production of LCOs, the so-called Myc-LCOs, produced by AM fungi (Maillet et al., 2011). LysM domains of bacterial proteins recognise peptidoglycan that contains a backbone alternating *N*-acetyl glucosamine and *N*-acetyl muramic acid. Appropriate changes to the module of three LysM domains that enabled recognition of an LCO molecule must therefore have been selected during evolution, and, for the rhizobium-legume (RL) symbiosis, there were presumably further selections for recognition of Nod factor decorations that are important in host specificity. Studies have identified critical residues in LysM2 of both MtNFP and LjNFR5. For MtNFP, this residue (L154) is different in orthologues of closely-related legume species nodulated by rhizobia producing different Nod factor structures (Bensmihen et al., 2011). Using complementation tests, the switch to L154 in the *Pisum sativum* NFP ortholog enabled *M. truncatula* mutant plants to be nodulated (Bensmihen et al., 2011). In LjNFR5, the L118 residue might define specificity towards decorations present on the non-reducing end of Nod factors (Radutoiu et al., 2007), and Nod factor binding to an individual LysM2 of LjNFR5 was reported (Sorensen et al., 2014). The sequence logos we generated with LysM domains of NFP proteins show that these two LysM2 residues implicated in the biological function of NFP proteins are both variable, compatible with direct or indirect roles in specific ligand recognition (**Figure 1C**). Using a sliding window approach in *L. japonicus*, some evidence was also provided for positive selection in LjNFR5 LysM2 (Lohmann et al., 2010). No significant signatures of selection were found in a study of nucleotide diversity of NFP in 30 genotypes of *M. truncatula* (De Mita et al., 2007).

During legume evolution, NFP became indispensable for the RL symbiosis, but not for the AM symbiosis, at least in model legumes (Ben Amor et al., 2003; Madsen et al., 2003). This suggests differences in activation of AM signalling in legume and non-legume plants. In addition, AM signalling activation might be different in monocots, despite the AM symbiosis appearing well before the monocot-dicot split, since there is no observable AM phenotype for *Osnfr5* knock-out mutants. However, these mutants show reduced symbiotic gene expression, and a chimeric receptor consisting of the extracellular domain of LjNFR5 and the intracellular domain of OsNFR5 complements an *Ljnfr5* mutant for rhizobial symbiosis, indicating some conservation of function (Miyata et al., 2016).

NFP and LYR3 are in different clades of the *M. truncatula* LysM-RLK family (Bono et al., 2018). Nevertheless, *NFP* genes are often in tandem with a *LYR3* gene, suggesting an ancient common evolutionary history, and a possible symbiotic role of the ancestral gene. This idea is reinforced by the finding that LYR3 proteins from *M. truncatula, L. japonicus* (LYS12)*, P. sativum, Glycine max, and Phaseolus vulgaris* are high affinity LCO binding proteins (Fliegmann et al., 2013; Malkov et al., 2016). Furthermore, Malkov et al. (2016) identified residues that are good candidates to be involved in a high affinity LCO binding site in LysM3, including a tyrosine residue, and their data suggest that loss of one or more of these residues in the non AM species *Lupinus angustifolius*, has led to loss of LCO binding. In our sequence logo analysis on LysM domains of LYR3 proteins (**Figure 1C**), this tyrosine residue is relatively well conserved across LYR3 proteins in plants, suggesting conservation of ligand binding properties. Although MtLYR3 can bind both Nod factors and Myc-LCOs, this protein is not indispensable for either symbiosis, but the LCO binding properties and reported interaction between MtLYR3 and MtLYK3 (Fliegmann et al., 2016) are suggestive of a symbiotic-related function.

**FIGURE 2:**
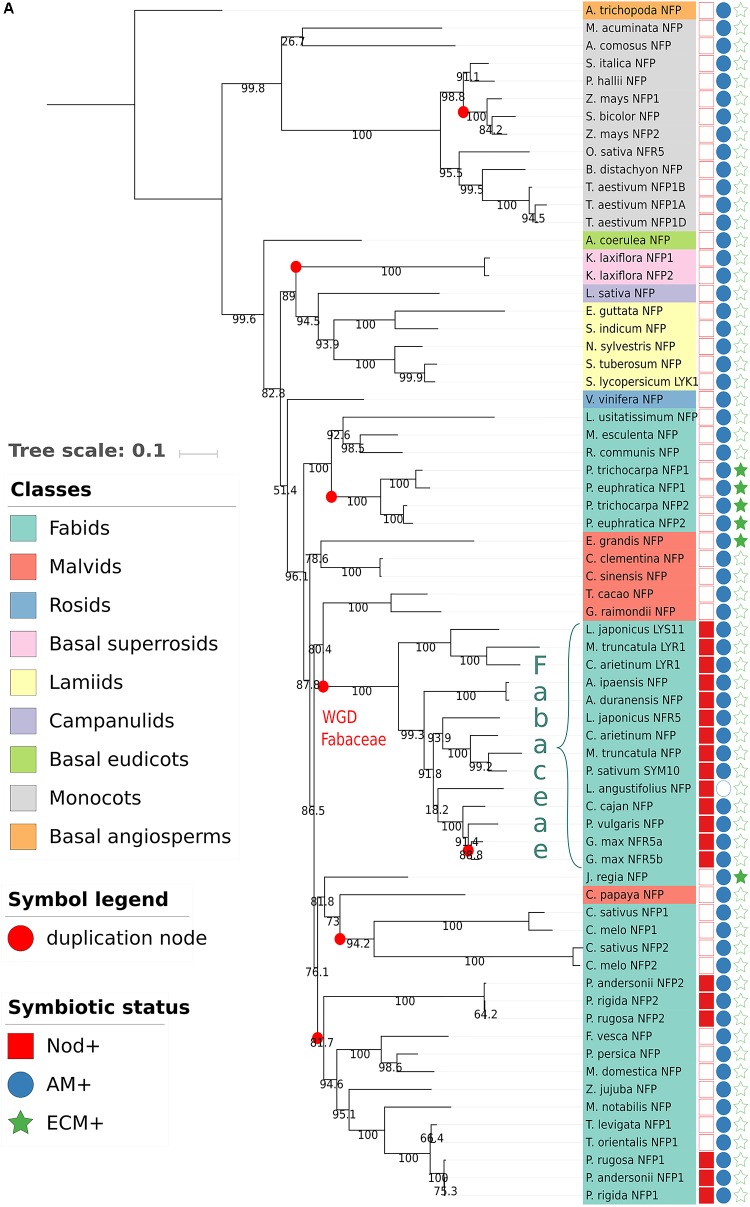

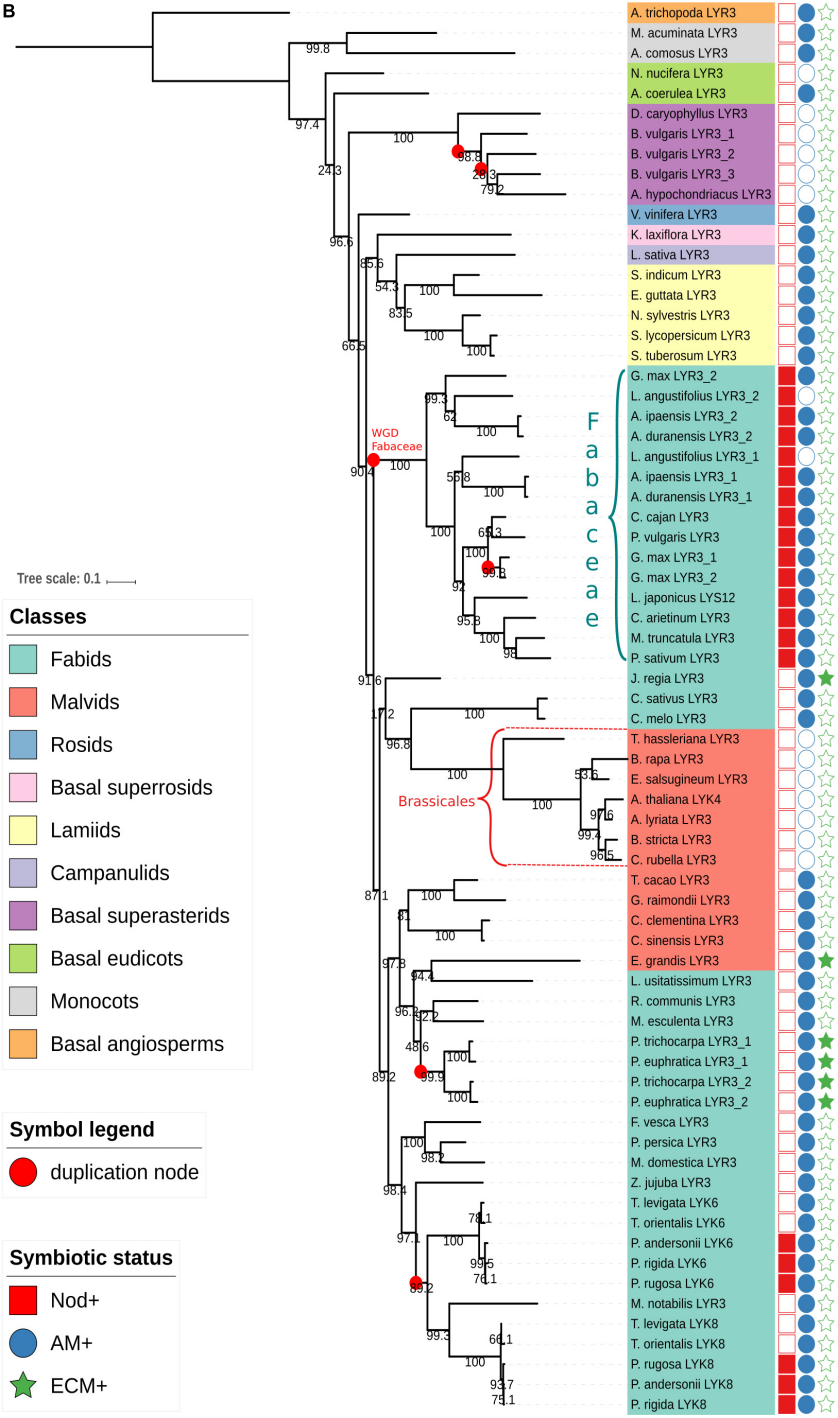
Phylogenetic trees generated from approximately 60 Angiosperm sequences of **(A)** NFP proteins or **(B)** LYR3 proteins (**Supplementary Table S1** and **Supplementary Data Sheet S1**). The sequences were aligned by Mafft (v7.271) (Katoh et al., 2002) with the following parameters: –maxiterate 1000 –retree 1 –genafpair. Sites with more than 50% of gaps were pruned from the alignments. The phylogenetic trees were computed with IQ-Tree (Nguyen et al., 2015) with the following parameters: -nt AUTO -bb 1000 -alrt 1000. The trees and metadata were visualised with Itol (Letunic and Bork, 2007). Branches with alrt support values (Anisimova and Gascuel, 2006) less than 0.75 were collapsed. Support values are displayed on the internal branches. The Amborella trichopoda LYR3 sequence was considered as an outgroup for the NFP tree, and the Amborella trichopoda LYR3 sequence was considered as an outgroup for the LYR3 tree. These proteins were chosen as outgroups as Amborella trichopoda is a basal Angiosperm species. An interactive view for the NFP tree is available at this url: http://itol.embl.de/tree/14799102217475161526387035. An interactive view for the LYR3 tree is available at this url: http://itol.embl.de/tree/14799102217474321526387017

To get further insights into the evolutionary histories of NFP and LYR3 proteins, we constructed phylogenetic trees with approximately 60 protein sequences each of NFP and LYR3 orthologues from 64 Angiosperm species (**Supplementary Table S1** and **Supplementary Data Sheet S1**). In the *Fabaceae* family, traces of the whole genome duplication event, dated at approximately 60 MYA (Cannon et al., 2015), can be seen (**Figure 2**). In some species the second copy of NFP (called LYR1 in *M. truncatula*) has been maintained (**Figure 2A**), while in other species, there is a second copy of LYR3 (called LYR3-2 in *Lupinus angustifolius*) (**Figure 2B**). No symbiotic role has been shown for any of these second copies, and divergence from consensus sequences could suggest a process of pseudogenisation. The presence of two copies each of NFP and LYR3 in *Populus* species could be due to the Salicoid genome duplication ancestral to speciation in this family, approximately 58 MYA (Harikrishnan et al., 2015). Other more recent events of genome duplication or hybridisation, have led to polyploidy in *G. max* and *Triticum aestivum*, respectively, and consequent additional copies of NFP and LYR3. Other examples of duplications include the two significantly different copies of NFP in both *Cucumis sativus* and *Cucumis melo*, and the three divergent copies of LYR3 encoded in tandem in *Beta vulgaris*.

A particularly interesting example of NFP duplication is in Rosales plants, where the *NFP2* gene was recently identified in three Parasponia species, and with high sequence divergence compared to the *NFP1* gene in these plants (van Velzen et al., 2018). Importantly, Van Velzen et al. found that NFP2 is absent in closely related non-nodulating Trema species (and also in other non-nodulating Rosaceae spp. like *Prunus persica*, *Fragaria vesca* and *Malus domesticus*), and that legume NFP proteins correspond to Parasponia NFP2. These data strongly suggest an importance of NFP2 for the ability of Parasponia, but not closely related plants, to nodulate. Furthermore, the absence of NFP2, as well as other key symbiotic genes, in Trema species, led van Velzen et al. (2018) to challenge the long-standing hypothesis on the evolution of nitrogen-fixing symbioses, pointing towards massive loss of nodulation rather than many events of parallel acquisition following a single hypothetical predisposition event in the common ancestor of the nitrogen-fixing clade.

It is also noteworthy that LYR3 was probably duplicated in a common ancestor of extant Rosales plant species, and two, divergent copies (LYK6 and LYK8) have been retained in both Parasponia and Trema species but not in other closely related species (van Velzen et al., 2018). Of these two proteins, LYK6 is more divergent between Parasponia and Trema species, suggesting different selection pressures on LYK6, but not LYK8, between these nodulating and non-nodulating plants. Taken together, ancestral events of gene duplication of NFP and LYR3 have usually been followed either by loss of one copy or by strong divergence of the two copies. In the case of preferential retention of both copies, which could have led to neofunctionalisation, NFP1 and NFP2 in Parasponia species are excellent candidates for such a scenario.

Consistent with the symbiotic roles of NFP proteins, plant species such as *Arabidopsis thaliana* that have lost the ability to establish the AM symbiosis, have lost NFP (Delaux et al., 2014). Exceptions to this rule are non-mycorrhizal lupin species, which have retained NFP to control the RL symbiosis. Thus in most *Brassicales* species, only LYR3 is present. However, in *Carica papaya*, which is a mycorrhizal species, LYR3 is absent, but NFP was found as reported before (Delaux et al., 2014). Also, only NFP was clearly found in Poaceae species such as rice, maize, and sorghum, but we found NFP and LYR3 in the monocots *Musa acuminata* and *Ananas comosus*, and in *Amborella trichopoda*, which is basal to both monocots and dicots.

Considering the trees of NFP and LYR3 proteins, each one has two clades in which sequences are phylogenetically close to each other and that are significantly phylogenetically distant from the other sequences in the tree. For NFP, these two clades correspond to *Fabaceae* and monocot proteins (**Figure 2A**), and for LYR3, they correspond to *Fabaceae* and *Brassicales* proteins (**Figure 2B**). This suggests that these groups of proteins have undergone significantly different evolutionary events, leading to specific and well conserved functions or properties. Nodulation is a likely explanation for the *Fabaceae* NFP proteins, while this divergence of *Brassicales* LYR3 proteins is correlated with absence of the AM symbiosis. Therefore, it is possible that the loss of the AM symbiosis was the driving force for a change of function for LYR3 in these plants. Since the *A. thaliana* LYR3 protein (called AtLYK4) controls chitin oligomer (CO) perception for immunity (Wan et al., 2012), a significant change to *Brassicales* LYR3 proteins could have been in terms of the ligand binding site predicted on LysM2 (Tanaka et al., 2013). The lack of LCO binding to LYR3 proteins from lupin species (Malkov et al., 2016) could also have evolved following loss of the AM symbiosis in these plants. LYR3 proteins of other non-AM species (*Dianthus caryophyllus*, *Beta vulgaris*, *Amaranthus hypochondriacus* and *Nelumbo nucifera*) do not group with the *Brassicales* LYR3 proteins, probably because of multiple, independent losses of the AM symbiosis in higher plants.

## The Evolution of Kinase-Active LysM-RLKs with Symbiotic Roles

LysM-RLK proteins with an active kinase domain also control symbiosis in legume and non-legume plants. OsCERK1 in *Oryza sativa* controls the AM symbiosis (Miyata et al., 2014; Zhang et al., 2015). OsCERK1 has a common ancestor with MtLYK3 and LjNFR1 that control nodulation in *M. truncatula* and *L. japonicus*, respectively (Gough and Cullimore, 2011). Phylogeny studies suggest that the common ancestor of MtLYK3 and LjNFR1 became neofunctionalised relatively recently following two rounds of legume-specific tandem duplications (De Mita et al., 2014). There is evidence of positive selection pressure for 3 amino acid residues in LysM1 of LYK3 (De Mita et al., 2014), compatible with data showing that MtLYK3 intervenes in controlling host range specificity (Limpens et al., 2003; Smit et al., 2007), and suggesting a role of LysM1 in Nod factor recognition. Similarly, Sulima et al. found evidence for strong selection pressure on LysM1 of MtLYK3, and also on LysM2 of a paralogous protein, MtLYK2 (Sulima et al., 2017). In *P. sativum*, evidence was found that PsLykX, which is encoded in a region syntenic to MtLYK2 and MtLYK3, is under positive selection in the extracellular region, mainly LysM1 again, compatible with a role in Nod factor recognition. This is reinforced by the higher allelic variation in PsLykX compared to highly homologous proteins, and one haplotype of PsLykX corresponds to nodulation specificity for a certain Nod factor structure (Sulima et al., 2017).

Chimeric protein studies indicate that the kinase “YAQ” sequence in LjNFR1 is important for activation of downstream symbiotic signalling leading to nodulation (Nakagawa et al., 2011). Phylogenetic studies suggest that the YAQ motif has an ancestral origin, predating the origin of nodulation (De Mita et al., 2014). Consistent with this and probably extending the role of the YAQ motif to the AM symbiosis, a very similar motif (YAR) is present in OsCERK1. Furthermore, the kinase domain of OsCERK1 combined with the LysM domains of LjNFR1 in complementation tests, can trigger nodulation signalling (Miyata et al., 2014). In legumes, PsLykX and MtLYK4, which are the results of recent duplications in *M. truncatula* and pea, respectively (Limpens et al., 2003; Sulima et al., 2017), are “YAQ-less” proteins that might heterodimerise with “YAQ-type” LysM-RLKs. The YAQ motif was also lost in AtCERK1 (De Mita et al., 2014), and this is reminiscent of the loss of NFP proteins and the divergence in LYR3 proteins in *Brassicales* plants.

In addition to LCOs, plant LysM proteins can recognise peptidoglycan and chitin (Antolin-Llovera et al., 2014), and these or other structurally-related, secreted or surface constituents of microbial symbionts are candidate ligands for symbiotic LysM-RLKs. COs are proposed to be symbiotic AM fungal signals (Genre et al., 2013), and OsCERK1 is involved in the recognition of such molecules, although no binding to chitin or COs has been observed (Shimizu et al., 2010; Shinya et al., 2012). For the RL symbiosis, the YAQ-less “LYK-type” LysM-RLK LjEPR3 was reported to bind rhizobial exopolysaccharide and control nodulation (Kawaharada et al., 2015). Interestingly, although there are candidate LjEPR3 orthologs in many plants, *Parasponia* spp. are notable exceptions (van Velzen et al., 2018), indicating that this protein is not indispensable for symbiosis.

## Symbiotic LysM-RLKs Can Also Control Immunity

Relationships between symbiosis and plant immunity are becoming more documented (Rey and Jacquet, 2018), and interestingly, both MtNFP and OsCERK1 have dual roles in symbiosis and immunity (Ben et al., 2013; Rey et al., 2013, 2015; Miyata et al., 2014; Zhang et al., 2015). In the case of OsCERK1 this is likely linked to CO perception (Carotenuto et al., 2017), and in legume plants LjLYS6/MtLYK9/PsLYK9 is a good candidate to have analogous roles to OsCERK1 (De Mita et al., 2014; Bozsoki et al., 2017; Leppyanen et al., 2018; our unpublished data). For MtNFP, one hypothesis is that it evolved to interact with both symbiotic and immune-related receptor proteins (Gough and Jacquet, 2013). Recently, LjLYS12 was reported to intervene in plant immunity (Fuechtbauer et al., 2018), suggesting that this LYR3 protein can also have a dual role in symbiosis and immunity.

More generally, people have asked whether the roles of LysM-RLKs in plant immunity diverged from symbiotic perception mechanisms or vice versa (Liang et al., 2014). Given that certain immune-type responses might be co-opted to facilitate symbiosis establishment (Limpens et al., 2015), then ancestral proteins could have had dual symbiotic and immune roles such that *Brassicales* LysM-RLKs have become subfunctionalised in immunity, while other proteins could have become subfunctionalised for symbiosis or retained the dual role. Indeed, the sequence divergence and biological roles of AtCERK1 and AtLYK4, can be interpreted as examples of subfunctionalisation in immunity.

## Future Challenges

Other symbiotic plant-fungal interactions include ectomycorrhiza (ECM), orchid mycorrhiza, ericoid mycorrhiza, and fine root endophytes have recently been described (Orchard et al., 2017). There are also other nitrogen-fixing symbioses, between Frankia bacteria and actinorhizal plants, and between certain legume plants and rhizobia that cannot produce Nod factors. Future studies should determine whether LysM-RLKs play roles in these diverse types of symbiosis. By analogy with the quenching role of the LysM protein Ecp6 of the fungal tomato pathogen *Cladosporium fulvum* (de Jonge et al., 2010) future investigations should also address whether any rhizobial or AM fungal LysM proteins have evolved symbiotic roles (Zeng et al., 2018). Evolutionary and structural studies are also needed to help decipher ligand binding properties of symbiotic LysM-RLKs, and more knowledge is needed on evolutionary events that have led to protein regulation, both quantitatively and at subcellular and tissue-specific levels.

## Sequence Files

**Glossary of LysM-RLK proteins discussed in the text:**

MtNFP: *Medicago truncatula* Nod Factor Perception, controls all Nod factor responses and nodulation, as well as plant immunity
LjNFR5: the *Lotus japonicus* ortholog of MtNFP
SlLYK10: the *Solanum lycopersicum* ortholog of MtNFP, controls the AM symbiosis
OsNFR5: the *Oryza sativa* ortholog of MtNFP
MtLYR3: *Medicago truncatula* high affinity LCO binding protein with unknown biological function
LjLYS12: the *Lotus japonicus* ortholog of MtLYR3
AtLYK4: the *Arabidopsis thaliana* ortholog of MtLYR3, controls immunity
MtLYK3: *Medicago truncatula* LysM receptor-like kinase3, controls rhizobial infection and nodulation
LjNFR1: the *Lotus japonicus* ortholog of MtLYK3
PslykX: a *Pisum sativum* LysM-RLK potentially involved in nodulation specificity for Nod factor structure
AtCERK1: *Arabidopsis thaliana* Chitin Elicitor Receptor Kinase1, controls plant immunity
OsCERK1: the *Oryza sativa* ortholog of AtCERK1, controls the AM symbiosis and plant immunity

## Author Contributions

CG and BL provided the sequences. CG, LC, BL, and J-JB analysed the sequences and wrote the text. LC constructed the phylogeny trees. CG and J-JB constructed the weblogos.

## Conflict of Interest Statement

The authors declare that the research was conducted in the absence of any commercial or financial relationships that could be construed as a potential conflict of interest.

## Supplementary Material

FIGURE S1 Sequence logos generated using WebLogo software (Schneider and Stephens, 1990; Crooks et al., 2004), and approximately 60 Angiosperm protein sequences of NFP for each individual LysM domain of NFP proteins.

FIGURE S2 Sequence logos generated using WebLogo software (Schneider and Stephens, 1990; Crooks et al., 2004), and approximately 60 Angiosperm protein sequences of LYR3 for each individual LysM domain of LYR3 proteins.

TABLE S1 Lists of NFP and LYR3 sequences used in **Figures 1**, **2** and **Supplementary Figures S1**, **S2**.

DATA SHEET S1 Protein sequences, whole protein, LysM and kinase domain alignments, and tree files.
