# Supplementary figures and images for: Evolutionary History of Plant LysM Receptor Proteins Related to Root Endosymbiosis
Source: Front Plant Sci. 2018 Jul 4;9:923. doi: 10.3389/fpls.2018.00923 (PMC6039847; doi:10.3389/fpls.2018.00923)

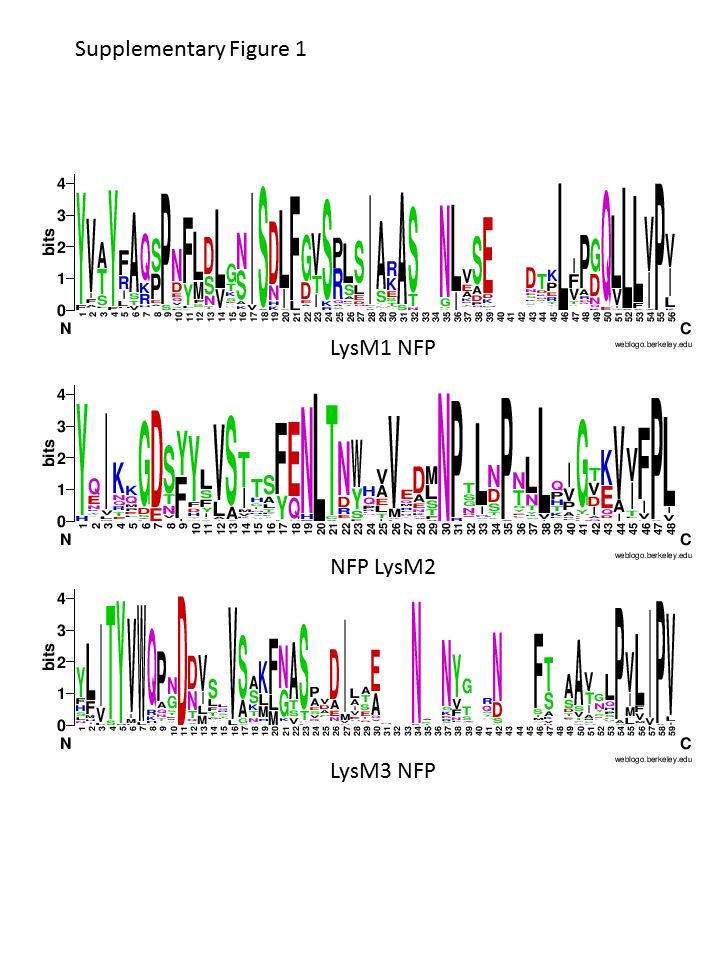

Supplement: FIGURE S1 — Sequence logos generated using WebLogo software (Schneider and Stephens, 1990; Crooks et al., 2004), and approximately 60 Angiosperm protein sequences of NFP for each individual LysM domain of NFP proteins. [file Image_1.TIF]

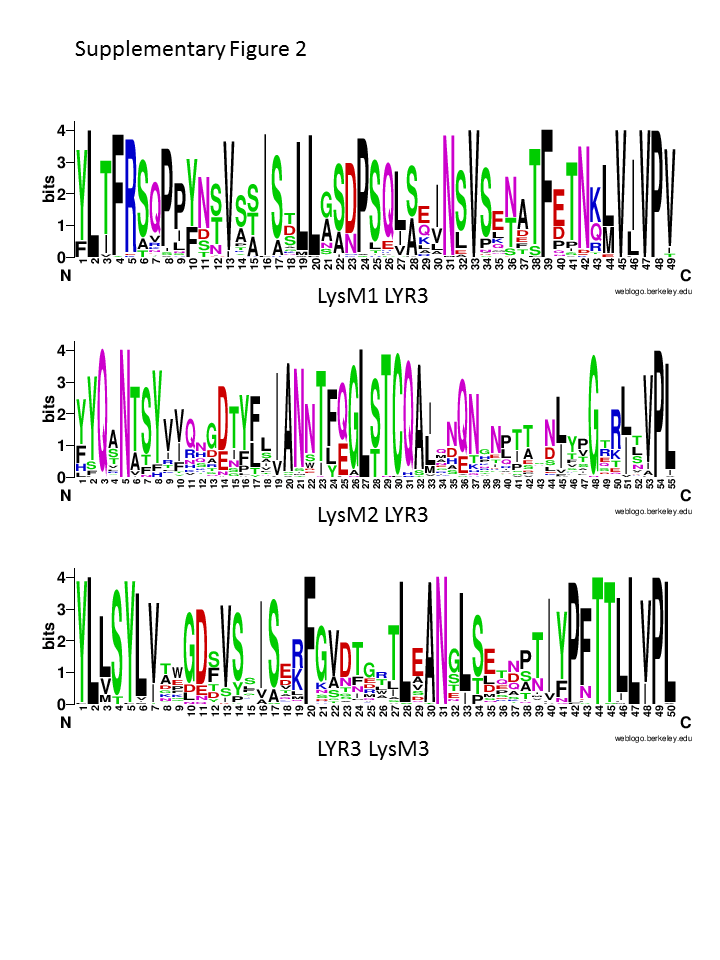

Supplement: FIGURE S2 — Sequence logos generated using WebLogo software (Schneider and Stephens, 1990; Crooks et al., 2004), and approximately 60 Angiosperm protein sequences of LYR3 for each individual LysM domain of LYR3 proteins. [file Image_2.TIF]
