# Supplementary material for: Evolutionary History of Plant LysM Receptor Proteins Related to Root Endosymbiosis
Source: Front Plant Sci. 2018 Jul 4;9:923. doi: 10.3389/fpls.2018.00923 (PMC6039847; doi:10.3389/fpls.2018.00923)
Supplement: DATA SHEET S1 — Protein sequences, whole protein, LysM and kinase domain alignments, and tree files. [file Data_Sheet_1.ZIP › Supplementary data/LYSM1-LYR3 alignment.docx]

Ma_LYR3_1 YLTFRSQIAYQSPVLIAYLLGADADNISRINGV-ATEFANVPNDQLVLVPV

Acom_LYR3 YLTFRALPPYDSVAAVSSLLAANPSLLSQSNSLPS-ASSAFPIGAKLSVPV

Csa_LYR3 FLTFRSRSPFNSVSSIATLLGSDPSELSRVNSV-N-ASATFPPDKLVLVPT

Cme_LYR3 FLTFRSRSPFNSVSSIATLLGSDPSELSRVNSV-N-ASATFPPEKLVLVPT

Adu_LYR3_2 YLTFKSQINYNSVPTISSLLNIDPTQLSKANSV-S-QNATFEINKLVIVPV

Aip_LYR3-2 YLTFESQINYNSVPTISSLLNIDPTQLSKANSV-S-QNATFEINKLVIVP-

Fv_LYR3 YLTFRSQPSYNTVTSIASMLASDPSQLAEINSV-S-ESATFATDKLVLVPV

LjLYR3 YLTFRTQPIYNSVYTISTLLSSDARHLAETMSV-S-QNTTFETNKLVIVPV

PsLYR3 YLTFRSQPIYSSVSTISSLLGSNPSQLAEINSV-S-VNETFEVNKMVIVPV

Ca_LYR3 YLTFRTQPIYNSISTISSLLGSNPSQLAEINSV-S-LNETFETNKMVIVPV

Mt-LYR3 YLTFRTQPIYSSVSTISSLLGSNPSQLAEINSV-S-LNETFETNKMVIVPV

Gm_LYR3_2 YLTFRSQPIYNSVKTISTLLGSDPSQLAKINSV-S-MNDTFETNKLVIVPV

Gm_LYR3_1 YLTFRSQPIYNSVKTISTLLGSDPSQLAKINSV-S-MNDTFETNKLVIVPV

Cca_LYR3 YLTFRSQPIYNSVSTISSLLGSDPSQLAKINSV-S-LNETFETNKLVLVPV

PvLYR3 FLTFRSQPIYNSVSTISTLLGSDPSQLAHINSV-S-LNDTFQTNKLVLVPV

Lan_LYR3_1 YLTFRSQPLYSSVSKISALLSSDPSQLAEINSV-S-LNGTFETNKLVIVPV

Adu_LYR3_1 YLTFRSQPLYSSVSTISSLLNSDPSQLALINSV-S-LNDTFEPNTLVIVPV

Aip_LYR3 YLTFRSQPLYSSVSTISSLLNSDPSQLALINSV-S-LNDTFEPNTLVIVPV

Lan_LYR3_2 YLTFRTQPLYNSVSSISKLLGSDPSKISEANSV-S-EEATFETNKLVIVPI

Md_LYR3 FLTFRSRAPYNAVSAISKLLASDPSPLAEVNSV-S-ETATFETNKLVIVPI

PpLYR3 YLTFRSQPPYNSVSAISAMLASDPSQIAEMNSV-S-ETATFETNKLVIVPI

Mn_LYR3 YLTFRSTPPYNTVIAISHLLSADPSEVSKINSV-S-KNSTFETNDLVFVPV

Ac_LYR3 YLIFRSIPPYNTVSSISGLLASDPIQLSRVNAV-S-GNQTFETNKEVIVPV

PanLYK6 YLTFRSQPPYDNVSAIADLLASDPSQISLVNLV-S-KNFTFETNRLVIVPV

Prig_LYK6 YLTFRSQPPYDNVSAIADLLASDPSQISLVNLV-S-KNFTFETNRLVIVPV

Prug_LYK6 YLTFRSQPPYDNVSAIADLLASDPSQISLVNLV-S-KNFTFETNRLVIVPV

Tlev_LYK6 FLTFRSQPPYDTVSAIADLLASDPSQISQVNLV-S-KNFTFETNRLVIVPV

Tori_LYK6 YLTFRSQPPYDTVSAISDLLASDPSQISQVNLV-S-KNFTFETNRLVIVPV

PanLYK8 YLTFRAVPPFNTVSAISDLLAANPSQVSEINLV-P-ETATFDTNKLVIVPV

Prig_LYK_8 YLTFRAVPPFNTVSAISDLLAANPSQVSEINLV-P-ETATFDTNKLVIVPV

Prug_LYK8 YLTFRAVPPFNTVSAISDLLAANPSQVSEINLV-P-ETATFDTNKLVIVPV

Tlev_LYK8 YLTFRAVPPFNTVSAISDLLAANPSQVSEINLV-P-ETATFDTNKLVIVPV

Tori_LYK8 YLTFRAVPPFNTVSAISDLLAANPSQVSEINLV-P-ETATFDTNKLVIVPV

Zj_LYR3 YLTFRSNPPYNNVSAISDLLASDPSQLSEINEV-S-ETETFDTNRLVIVPV

Me_LYR3 YLVFRSQPPYTSVASISTLLTSDPSELAAINSV-S-ETASFDTNKLVIVPV

Rc_LYR3 YLTFRSQPPYTNVTSISTLLNSDPSQLSAINSV-S-ETATFDTNKLVIVPV

Egut_LYR3 YLTFRSTPPYDTLSAVSALFSVNSSQLAQLNSVPETNTAVFEADRMVLIPV

Si_LYR3 YLTFRAQPPYDSVATISTLLAANSSQLAQLNSV-P-ENALFDTNRMVLVPV

PinLYR3 YLTFRSQPPFNSIATISGLLGADPLQLAQINSV-S-NSATFETNQMVLVPV

PaxLYR3 YLTFRSQPPFNSIATISGLLGADPLQLAQLNAV-S-NSATFETNQMVLVPV

St_LYR3 YLTFRSQPPFNTVPSISSLLGANPSQLSQLNSV-S-QNATFNTNQMVLVPV

Sl_LYR3 YLTFRSQPPFNTVSSISSLLGANPSQLSQLNSV-S-QNATFNTNQMVLVPV

Nb_LYR3 YLTFRSQPPFTSVASISSLLGADPLELSQLNSV-A-QNATFDTNQMVLVPV

Lu_LYR3 YLTFRAQPPYTTVASISTLLGSNPSQLAAINSVVP-ETSSFPTNQLVLVPV

Pt_LYR3_2 YLIFRSQPPYNTVASISTLLGSDPSQLSEVNSV-S-ETTSFPSNQLVIVPV

Pe_LYR3_2 YLIFRSQPRYNTVASISTLLGSDPSQLSEVNSV-S-ETTTFPSNQLVIVPV

Pt-LYR3_1 YLIFRSQPPYSTVASISTLLGSDPSQLSQINSV-S-ETTSFPTNQLVLVPV

Pe_LYR3_1 YLIFRSQPPYTNVASISTLLGSDPSQLSQINSV-S-ETTSFPTNQMVLVPV

Eg_LYR3 YLIYRSNPPYDTVRSIAKLLASDPSQLSAINSV-G-PNATFPTNNMVIVPV

Ccl_LYR3 FLIFRSKPPFNTVASLRFWPLSH-LSSPKINSV-S-ETATFETNQMVIVPV

Vv_LYR3 FLIFRSEPPYNDVSSISDLLGSDPSQLAQINSV-D-ETATFETKKEVIVPV

Csi_LYR3 FLIFRSKPPFNTVASISTLLASKPSQLSKINSV-S-ETATFETNQMVIVPV

Gr_LYR3 YLVFRSQPLFNNVTSISNLLSSDPSQIAAINEV-S-ETATFQTNQMVIVPV

Tc_LYR3 YLIFRSQPSFNTVASISNLLSSDPSQIAEINEV-S-ETASFETNQMVIVPV
