# Supplementary material for: Evolutionary History of Plant LysM Receptor Proteins Related to Root Endosymbiosis
Source: Front Plant Sci. 2018 Jul 4;9:923. doi: 10.3389/fpls.2018.00923 (PMC6039847; doi:10.3389/fpls.2018.00923)
Supplement: DATA SHEET S1 — Protein sequences, whole protein, LysM and kinase domain alignments, and tree files. [file Data_Sheet_1.ZIP › Supplementary data/LYSM1-NFP alignment.docx]

Ma_NFP FVLYRTQP-EYTDLGSISDLFGVSRLSLEEAN--DLRSE---EVTLPADQLLVVPV

Egut_NFP YITYRVKPP-YSNLGAISDLFGISRLDISTAT--NLASE---DARLIPDQILLIPI

Si_NFP YVTYRARQP-YSDLGSISDLFGISRMDIASAT--SLTSE---NHSLFPDQLLLIPI

Acom_NFP YVAYRTQLPNYADIGAISDLFGVSRLSIAKAN--NLSSE---DGTLLPDQLLLVPV

Pax_NFP YVTYRARPP-YLDVGSISDLLGVSRLNIAKAT--GLASE---DTELFPDQLLLVPV

Pin_NFP YVTYRARPP-YLDVGSISDLLGVSRLNIAKAT--GLASE---DTELFPDQLLLVPV

Nb_NFP YVTYRARPPNYFDVGSISDLLEVSRLSIAKAT--GLVSE---DTELFPDQLLLVPV

St_NFP FISYRARPPNYLDVGSISDLLEVSRLSIATAT--GLASE---DTELFPDQLLLAPV

Sl_NFP FISYRARPPNHLDVGSISDLLEVSRLSVATAT--GLASE---DTELFPDQLLLVPV

Csa_NFP_2 YVTFFAKSPDFLDLESVSDLFGVRPSLIADAS--NLNAE-DGRRDLFPGELLLIPV

Cmel_NFP1 YLTFFAKSPDFLDLDNVSDLFGVRRSLIADAS--NLNAE-NGR-RLFPGELLLIPV

Mt_LYR1 YVAYFANSPNFLTLTAISDIFDTSPQSIARAS--NIKDE---NMNLIHGQLLLIPI

Lj_LYS11 YVTYFAQSPNFLTLTSISDLFDTSPLSIARAS--NIKDE---NQNLVPGQLLLVPI

Ca_LYR1 YAAYHAHSPNFLTLTSISDIFDTSPLSIARAS--NIKDE---NKELIPGQLLLVPI

Lan_NFP YVTYIAHSPNFLSVVSISNIFDTSPLSIARAS--NLEAE---DNTLIPDQVLLVPV

Cca_NFP YVTYIAQSPNFLSLTSISNLFDTSPLSIARAS--NLDPD---NNKLIPDQILLIPV

Pv_NFP YVTYISQSPNFLSLTSVSNIFDTSPLSIARAS--NLQHE---EDKLIPGQVLLIPV

Gm_NFR5a YVTYIAQSPNFLSLTNISNIFDTSPLSIARAS--NLEPM---DDKLVKDQVLLVPV

Gm_NFR5b YVTYIAQSPNFLSLTSISNIFDTSPLSIARAS--NLEPE---DDKLIADQVLLIPV

Mt_NFP YVAYRAQSPNFLSLSNISDIFNLSPLRIAKAS--NIEAE---DKKLIPDQLLLVPV

Aip_NFP YVTYIAKSPNFLSLSNISDIFDTSPLSIARAS--NIKNE---GDKLVPGQVLLIPV

Adu_NFP YVTYIAKSPNFLSLSNISDIFDTSPLSIARAS--NIKNE---GDKLVPGQVLLIPV

Ps_SYM10 YVTYFARSPNFLSLTNISDIFDMSPLSIAKAS--NIEDE---DKKLVEGQVLLIPV

Lj_NFR5 YVTYTAQSPNLLSLTNISDIFDISPLSIARAS--NIDAG---KDKLVPGQVLLVPV

Ca_NFP YVTYIAQSPNFLSLTNISDLFDISPLSIARAS--NIDDE---DKELIPGQVLLVPV

Csa_NFP_1 YVSYFTKSSQFFDLHSISKLFGVKALKIAKAS--NLESD---KTPLFDGQLLFIPV

Cmel_NFP2 YVSYFAKSSQFFDLHSISKLFGVKALEIAKAS--NLESD---KTPLFDGQLLFIPV

Mn_NFP YVAYFAQEPDFMDLKSISDLFGVKSSLISEAS--NLVTE---TTKLIPGQLLLVPV

Prig_NFP1 YVAYFAQPPLFMDLKSISN-FGVSPSSISEAS--NLVSE---STKLTRGQLLLIPL

Tori_NFP1 YVAYFAQPPVFMDLKSISNLFGVSPSSISEAS--NLVSE---ATKLTPGQLLLIPL

Tlev_NFP1 YVAYFAQPPVFMDLKSISNLFGVSPSSISEAS--NLVSE---STKLAPGQLLLIPL

Pan_NFP YVAYFAQPPLFMDLKSISNLFGVSPSSISEAS--NLVSE---STKLTRGQLLLIPL

Prug_NFP1 YVAYFAQPPVFMDLKSISNLFGVSPSSISEAS--NLVSE---STKLTRGQLLLIPL

Fv_NFP YVAYFVESPGYMNLENISDLFGVSVSSISQAS--NLASSYTGQTRLVAGQLLLVPI

Pan_LYK7 YVAYFSQPPNYMNVGNISDLFGISQALIAKSS--NLVSK---DSPLIPQQLLLIPL

Prig_NFP2 YVAYFSQPPNYMNVGNISDLFGISQALIAKSS--NLVSK---DSPLIPQQLLLIPL

Prug_NFP2 YVAYFSQPPNYMNVGNISDLFGISQALIAKSS--NLVSK---DSPLIPQQLLLIPL

Pp_NFP YVSYLAQ-PQFLSLGNISDLFGVSSLSITKAS--NLVSE---QIRLIAGQLLLVPI

Md_NFP YVSYFAR-PQFMSLENISHLFGVSPLSIAKAS--NLVSE---HIRLIAGQLLLVPI

Atr_NFP YVLYRAQVPEFSDLGNISDLFGVSRLSIMRAS--NLTAE---VVTLSQNQQVFVPI

Tc_NFP YAAYFAQEPDFLDLQKISDLFGTSPPETARAS--NLASV---DTQLFPGQLLLVPI

Gr_NFP YTAYFAQEPDYLDLQNIAKLFGTSPQEIARAS--NLVSE---DTRLFPGQLLLVPI

Eg_NFP YVAYFAQPPDFMDVGNISDLFHVSRRSIIQAS--NLTSE---YNGLVQGQLLLVPI

Zj_NFP YVAYFAKPSNFMSLDNISDLFMVSRLSIAKAS--NLISE---YTKLVPGQLLLIPI

Ac_NFP YVVYRAQSPDFLYLGDISDLFGVSRSDIKKAS--NLVSE---EFQLVPDQLLLVPI

Vv_NFP YVIYRAQAPGFLDVGNISDLFGISRLSIAEAS--NLASE---EARLSPDQLLLVPI

Lu_NFP YVSYHVQSPNFLNLGNISDLFGVSRLSIATAS--NLASE---DSPLVLDQLLLVPI

Ccl_NFP YVAYFAQSPEFLDLSNISDLFGVSRLSIARAS--NLVSE---DTPLVPKQLLLVPI

Csi_NFP YVAYFAQSPEFLDLSNISDLFGVSRLSIARAS--NLVSE---DTPLVPKQLLLVPI

Me_NFP YVAYLAQSPNFLNLGNISDLFAVSRLAIASAS--NLVSE---DTSLIPDQLLLVPV

Rc_NFP YVAYYAQPPNFLNLGNISDLFAVSRLSIASAS--NLVSE---DIPLMPNQLLLVPI

Pt_NFP_2 YFTYLAQPPNFLDLGNISDLFGVSRKEIATAS--NLESE---DTPLFPNQLLLVPK

Pe_NFP2 YFTYLAQSPNFLDLGNISDLFGVSRKEIANAS--NLESE---DTPLFPNQLLLVPK

Pt_NFP_1 YISYLAQPPDFLDLGKISHLFGISRTLIASAS--NLVSE---DTPLFPNQLLLVPI

Pe_NFP1 YFSYLAQAPDLLDLGKISHLFGISRTLIASAS--NLVSE---DTPLFPNQLLLVPI
