# Supplementary material for: Evolutionary History of Plant LysM Receptor Proteins Related to Root Endosymbiosis
Source: Front Plant Sci. 2018 Jul 4;9:923. doi: 10.3389/fpls.2018.00923 (PMC6039847; doi:10.3389/fpls.2018.00923)
Supplement: DATA SHEET S1 — Protein sequences, whole protein, LysM and kinase domain alignments, and tree files. [file Data_Sheet_1.ZIP › Supplementary data/LYSM2-LYR3 alignment.docx]

Atr_LYR3 YSQANITYQIQKGNTYYLVSTLQFLNLTTFQAVIVANPSLVPTNLTIGVEVVFPI

Eg_LYR3 HYQANATYSIRRGDNYYVIVNDIYQGLTTCRAIKDQNDIQ-TVDLYSGD-LTIPI

Csi_LYR3 HYQANTTYFVQNGDTYFLIANNTFQGLSTCQALQDQHGNV--SNFGVGTRLLAPL

Ccl_LYR3 HYQANTTYFVQNGDTYFLIANNTFQGLSTCQALQDEHGNV--SNFGVGTRLLAPL

Adu_LYR3_1 YYQS--NYVYHNAETYFLIANNTFQGLTTCQAMMHQNANL--SNLYPGRQLAVPL

Aip_LYR3-1 YYQS--NYVYHNAETYFLIANNTFQGLTTCQAMMHQNANL--SNLYPGRQLAVPL

Lan_LYR3_1 YYQANTSYVVQNADTPFLIASNTFEGLSTCQALQNENHNP--WNMFQGRELLVPL

LjLYR3 YYQANTSYAFQNTDTPFSIANNTFEGLTTCQALMHENHNP--GHLYLGRELTVPL

PsLYR3 YYQANTSYVFQNTDTYFLVANNTFEGLSTCQALMHENHNP--ADIYPGRELLVPL

Ca_LYR3 YYQANTTYVFQNTDTYFLVANNTFEGLSTCQALMNQNHNP--GDVYPGRKLLVPL

Mt_LYR3 YYQANTSYVFQNTDTYFIVANNTFEGLSTCQALMHENHNP--GDVYPGRKLLVPL

Cca_LYR3 YYQTNTSYVFQNAETYFLIANNTFEGLTTCQALEHQNRNP--ADIYPGRKLLVPL

PvLYR3 YYQTNTSYVFQNSETYFLIANNTFEGLTTCQALQSQNHNP--ANIYPGRKLLVPL

Gm_LYR3_2 YYQTNTSYEFHNSETYFLIANNTFEGLTTCQALENQNHNP--ANIYPGRRLLVPL

Gm_LYR3_1 YYQTNTSYVFQNSETYLLIANNTFEGLTTCQALENQNHNP--ANIYPGRRLLVPL

Adu_LYR3_2 YYQYNTSYKVQSGDNYFIIANNTFEGLSTCQAMKDQNKID-ELKLSPGDKLRVPL

Aip_LYR3-2 YYQYNTSYKVQSGDNYFIIANNTFEGLSTCQAMKDQNKID-ELKLSPGDKLRVPL

Lan_LYR3_2 YYQFNTSFIVQQDDNYFVIANNTFEGLSTCQALQDQNRIP-DVDLDVGTKLVVPL

Lu_LYR3 YYQTNTSFVVRTGDNYFFIANQTLQGLSTCQAIRNGNRRS-TTNIFPGQTLDVPL

Me_LYR3 YSQANTSYVVQAGDSVFLIANNTYQALSNCQAVQSQNRMQ-DFDIVTGERLTIPL

Rc_LYR3 YYQANTSYVVQAKDAPFFIANNTFQGLSTCQAINDQNRRQ-TVDIFPNEILHIPL

Ma_LYR3 YYQHNVSYTLTSRDTYFIVANDTYQGLSTCQALIAQNPYG-SLNLTAGLRVDVPL

St_LYR3 FYQSNASYVIRRDDTFLNVAINTLQGLSTCHAINAENSEQ-ANNLVVGSRINVPL

Sl_LYR3 FYQSNASYVIRRDDSFLNIAMNTLQGLSTCQAINAENSEQ-ANNLVVGSRINVPL

Nb_LYR3 YYQTNTSYVIKKDDNFLTIANNTLQGLSTCHAINAENKEQ-ANNLILGSRINVPL

PinLYR3 YYQTNTSYVIRKDDTYLTIANNTLQGLSTCQAINDQNKEQ-ESSLVLGSRINVPL

PaxLYR3 YYQTNTTYVVRKDDTYLTIANNTLQGLSTCQAINDQNKEQ-ESSLVPGSRINVPL

Ac_LYR3 YYQVNASYVIEHDDTYLAIANNTYQGLSTCQALRNQNTLT-TKNLYSGTRITVPI

Egut_LYR3 HYQVNASYVVRQDDTYFGIANATFQGLSTCQSLETQNGDP-TRYIFPGSRITVPL

Si_LYR3 HYQVNASYVVQQGDTYFMIANNTFQGLSTCQTLQAENDTL-TRNLYAGTRITVPL

Vv_LYR3 FSQANTSYVVQHGDTYLLIANNTFEGLSTCQALRSQRTSL-TTNIYTGTKLTVPL

Pe_LYR3_2 YSQANASYIVQPNDTLLLIANNTYQGLSTCQALQNQKSTR-TDDILSGETLTVPL

Pt_LYR3_2 YSQANASYIVQPNDTLFLIANNTYQGLSTCQALQNQKTTR-TDDILSGETLTVPL

Pe_LYR3_1 YFQANASYIVQSGNTPFLIANNTYQGLSTCQAIRDQKGTG-TVDIFAGETLTVPL

Pt_LYR3_1 YFQANASYIVQSGNTPFLIANNTYQGLSTCQAIRNEKGTR-TVNIFAGETLTVPL

Csa_LYR3 FFQSNVSFTTRTGDSYFVIANETLQGLSTCQSLISQNPNVSVTSIKGGERILVPL

Cme_LYR3 FLQSNVSFTTRTGDSYFAIANETLQGLSTCQSLISQNPNIGVTSIKGGERILVPL

Prig_LYK_8 FYQSNTFYRVNQREIYTAIANWIFEGLTTCQAIANQTGNPIAQNLTVGQRISVPL

Prug_LYK8 FYQSNTFYRVNQREIYTAIANWIFEGLTTCQAIANQTGNPIAQNLTVGQRISVPL

PanLYK8 FYQSNTFYRVNQREIYTAIANWIFEGLTTCQAIANQTGNPIAQNLTVGQRISVPL

Tlev_LYK8 FYQSNTFYRVNQGEIYTSIANWIFEGLTTCQAIANQTGNPIAQNLTVGQRISVPL

Tori_LYK8 FYQSNTFYRVNQGEIYTSIANWIFEGLTTCQAIANQTGNPIAQNLTVGQRISVPL

Mn_LYR3 FYQRNASYIIKSGDTYLALANNTFQGLTTCQAFANQTGNPPPLELETGQRIVVPL

Gr_LYR3 HYQRNTSYIIQSGDGYFLIANSTFQALSTCQAIQNQQPVIPSESLTPGMRITVPV

Tc_LYR3 YYQVNTTYTVQSGDGYFSIANNTFQALSTCQAIQNQQPDIPSQSLTIGLRITVPL

Zj_LYR3 YYQRNTSYVIKEAAGYLFIANNTLQGLTTCQAIANQNPGLTSSNLSIGARLNAPL

Tlev_LYK6 LYQANTSYVIQHGDTFLSIANSTFQGLSTCQAIGNQNNNLTTSLLYTGTRISVPL

Tori_LYK6 LYQANTSYVIQHGDTFLSIANSTFQGLSTCQAIGNQNNNLTTSLVYTGTRISVPL

PanLYK6 LYQANTSYVIQHGDIFLSIANSTFQGLSTCQAIGNQNNNLTTRLLYTGTRISVPL

Prig_LYK6 LYQANTSYVIQHGDIFLSVANSTFQGLSTCQAIGNQNNNLTTRLLYTGTRISVPL

Prug_LYK6 FYQANTSYVIQHGDIFLSVANSTFQGLSTCQAIGNQNNNLTTRLLYTGTRISVPL

Md_LYR3 YYQLNTSHVVVNGDTFFIIANNTLQGLSTCQAMLNQNGNRTAEDLSIGDRLNVPL

Fv_LYR3 YYQLNTSHVVVHGDTFLVIGNNTFQGLSTCQAIMKQNSNLTTKNLYTGTTLTIPL

Pp_LYR3 FYQLNTSHVVVHGDTYFVIANNTLQGLSTCQAMMNQNTNLTTKELYSGTRLSVPL
