# Supplementary material for: Evolutionary History of Plant LysM Receptor Proteins Related to Root Endosymbiosis
Source: Front Plant Sci. 2018 Jul 4;9:923. doi: 10.3389/fpls.2018.00923 (PMC6039847; doi:10.3389/fpls.2018.00923)
Supplement: DATA SHEET S1 — Protein sequences, whole protein, LysM and kinase domain alignments, and tree files. [file Data_Sheet_1.ZIP › Supplementary data/LYSM2-NFP alignment.docx]

Mt_NFP YSIKQGDNFFILSITSYQNLTNYLEFKNFNPNLSPTLLPLDTKVSVPL

Pan_NFP2LYK7 YQVEPGDTYHYLSTLLFENLTNSQVMKKMNPEISPEYVLPYIDIIIPV

Prig_NFP2 YQVEPGDTYHYLSTVLFENLTNSQVMIQMNPEISPEYVLPYIDIIIPV

Prug_NFP2 YQVEPGDTYHYLSTVLFENLTNSQVMIQMNPEISPEYVLPYIDIIIPV

Mt_LYR1 HLIKESESYYYLSTISYQNLTNWQTVEDSNPNLNPYLLKIGTKINIPL

Ca_LYR1 HFIKQGESYYYLSTISFQNLTNWQTVEDSNPNLNPYLLKLGTKIVIPL

Lj_LYS11 HMIKEGESYYYLSTTSYENLTNWETVQDSNPNYNPYLLPVGIKVVIPL

Eg_NFP YEIMKGDSYYLVSTHAFENLTNWQTVEAANPSLNPNTLKAGTRVVFPL

Acom_NFP YEIAKGDSFYLVSIRAFENLTDYNAVEEFNPSLDPSHLKVGQEVIFPL

Fv_NFP YSIKRGDSYYVVSMYTFENLTRWPLVVEMNPALVPSLLQIGVKVIFPL

Mn_NFP YEIKMGDSYFIVSRYFFENLTDWHVVLVTNPSLNPNLLKIGTKVIFPL

Pan_NFP1 YNITMGDSYYLVSIHSFENLTNWPLVRDTNPTLNPNLLQIGTKVIFPL

Prig_NFP1 YNITMGDSYYLVSIHSFENLTNWPLVRDTNPTLNPNLLQIGTKVIFPL

Tlev_NFP1 YNITMGDSYYLVSIHSFENLTNWPLVRDTNPTLNPNLLQIGTKVIFPL

Tori_NFP1 YNITMGDSYYLVSIHSFENLTNWPLVRDTNPTLNPNLLQIGTKVIFPL

Prug_NFP1 YNITMGDSYYLISIHSFENLTNWPLVRDTNPTLNPNLLQIGTKVIFPL

Md_NFP YEIKSGDSFYLVSINSFENLTDWHEVLNMNPTLDPSLLQIGQKVIFPL

Pp_NFP YEIKKGDNYYLVSINSFENLTNWHAVLDMNPTLDPTLLQIGVKVTFPL

Ccl_NFP YQIKKGDSYYLVSITSFENLTNWHAVREMNPGINPNLLQIGVKVTFPL

Csi_NFP YQIKKGDSYYLVSITSFENLTNWHAVLEMNPGINPNLLQIGVKVTFPL

Csa_NFP_1 YKINQGDTFYLVSTSFFEHLCDSDIVVKMNPSLNPNNLSVGVEAVFPL

Cmel_NFP2 YKINKDDTFYLVSTSFFENLCDSDIVVKMNPSLNPNNLSVGVEAVFPL

Ma_NFP YTIKEGDNFFLVSTNAFGNLTDYHLVEDLNPTLEPTSLRPGQEVIVPV

Atr_NFP YQIQKSDSFYLVSTYKFENLTDYQAVGDLNPNLNPINLWVGTEVIFPI

Ac_NFP YQIKKGDSFYLVSVHAFENLTNYHVVMDMNPTLNPTLLQIGTQVIFPI

Pax_NFP YQIKKGESFYSASIGAFENLTNYHVVQDMNPTLDPTNLTVGTEAVFPL

Pin_NFP YQIKKGESFYSASIGAFENLTNYHVVQDMNPTLDPTNLTVGTEAVFPL

St_NFP YQIKKGDNFYSVSIGAFENLTNYHVVQDMNPTLDPTNLTIGAEAVFSL

Sl_NFP YQIRKGDSFYSVSIRAFENLTNYHVVQDMNPTLDPTNLTIGAEAVFPL

Nb_NFP YQIKKGDSFYSVSVGAFENLTDYHVVQDMNPTLDPTNLTVGAEAVFPL

Lj_NFR5 YQIQLGDSYDFVATTLYENLTNWNIVQASNPGVNPYLLPERVKVVFPL

Aip_NFP YELRQGDMYDFVSKTTYENLTNWRAVNDSNPDLNPVLLPVGVKVLFPL

Adu_NFP YELRQGDVYDVVSKTTYENLTNWRAVNDSNPDLNPVLLPIGVKVLFPL

Pv_NFP YEINQGDSFYFVATTLYQNLTNWHAVMDLNPGLSPFTLPIGIQVVIPL

Cca_NFP YEINQGDSFYFVATTLYQNLTNWHAVMDLNPGLSPFTLPIGIQVVIPL

Gm_NFR5a YEINQGDSFYFVATTSYENLTNWRAVMDLNPVLSPNKLPIGIQVVFPL

Gm_NFR5b YEINPGDSFYFVATTSYENLTNWRVVMDLNPSLSPNTLPIGIQVVFPL

Ps_SYM10 YTIKLGDNYFIVSTTSYQNLTNYVEMENFNPNLSPNLLPPEIKVVVPL

Ca_NFP YTIKLGDSYILVSTTSYQNLTNYLEMEDSNPGLNPNLIPPFIKVVVPI

Lan_NFP YVIKLGDTYELVVETIYEHLTNWLVVADLNPGLVPTLLTVGVEVIFPL

Zj_NFP YQIKKDDSFYSVSTGEFQRLTEWHVVEDLNPTLNATFLQIGVEVVFPL

Egut_NFP YRIKKEDSFYSVSTRPFQNLTNFITVEETNPTMNPNNLTIGVEAVFPL

Si_NFP YRIKKDDIFYSVSIKPFQNLTNYYVVEEMNPMLNPNNLTIGVEVVFPL

Tc_NFP YNIKPGDTFYIVSTTTFENLTNYTAVEDMNPDRDPRKLKVGDKVVFPL

Gr_NFP YDIKFGDTYYIVSTTVFEHLTNFTTVGDMNPSLDPKSLQVGDKVVFPL

Csa_NFP_2 YQIKEGDVYYTLAMTSFQNLTEWHVVNASNPNLDPNLLHKGDEVTFPL

Cmel_NFP1 YQIKEGDVYYTLAMTSFQNLTEWHLVNASNPNLGPSLLHKGDEVTFPL

Vv_NFP YKIKTDDSFYFVSVTVFENLTNYNAVEALNPGLEPTTLQVGVEVVFPL

Lu_NFP YQIKGGDNFYLVSITSLENLTNWQVAELLNPDLPPTLLHPGDHVVLPI

Rc_NFP YQIKPGDSFYFVSTTYFENLAKWQAVESFNPNLDPTLLHPGDKVVFPL

Me_NFP YQIKHGDNYYLVSTTSFENLTNWEAVKVLNPKLDPNLLHPGDKVIFPL

Pt_NFP_2 YQIQQGESFYLVSTTSFENLTRWQEVEALNPSLTPTLLHAGDKVIFPL

Pe_NFP2 YQIRQGESFYLVSTTSFENLTRSQEVEALNPSLTPTLLHAGDKVIFPL

Pt_NFP_1 YQIQQGDSIYSVSTISFENLTRWQEVEALNRSLTPTLLHAGDEVIFPL

Pe_NFP1 YQIQQGDSIYSVSTISFQNLTRWQEVEALNRGLTPTLLHAGDKVIFPL
