# Supplementary material for: Evolutionary History of Plant LysM Receptor Proteins Related to Root Endosymbiosis
Source: Front Plant Sci. 2018 Jul 4;9:923. doi: 10.3389/fpls.2018.00923 (PMC6039847; doi:10.3389/fpls.2018.00923)
Supplement: DATA SHEET S1 — Protein sequences, whole protein, LysM and kinase domain alignments, and tree files. [file Data_Sheet_1.ZIP › Supplementary data/LYSM3-LYR3 alignment.docx]

Gr_LYR3 YLLSYPVAEGETVSSISALFGEDPERTLEANQLPDPSSTVFFETSLLVPL

Tc_LYR3 YLLSYLIAEGDSVPGISQLFGADTAKTLEANELSDPTINFF--TTLLVPL

Zj_LYR3 YLMSYLVAEGDQVSLISQRFGADTKLTSEANSLPNTNINPF--TTFLVPL

Ma_LYR3 YLLTYLITWGDDVPTIADRFHADYQAVLHANNISS-SSTIYPFTTLLVPL

Sl_LYR3 YLLTYLIASGEFVSFISDKFGVDFRATLAANSIPEDAPTVFPNTTLLVPL

St_LYR3 YLLTYLIASGEFVSFISDKFGVDFRATLAANSIPEDAPTVFPNTTLLVPL

Si_LYR3 YLLSYLITWNQFVSGISALFGVDTGRTLAANGLSESS-IVYPFTTLLVPL

Egut_LYR3 YLLSYLVTWNQFVSSISALFGVDTGQTLAANTLSETDFNIYPFTTLLIPL

PaxLYR3 FLLSYLVTFGEFVSLISDKFGAGTGATLAANGLSETDTNIYPFTTLLVPL

PinLYR3 YLLSYLVTFGEFVSLISEKFGAGTGATLAANGLSETDTNIYPFTTLLVPL

Nb_LYR3 YLLSYLVTFGEFVSLISDKFRVDTRATLAANGLSENDATIFPFTTLLVPL

Eg_LYR3 YLLSYVVAEGNYVGTISGRFGTNTAWTLEANGLSEQVSTIYPFTTLLVPV

Me_LYR3 YLLSYLITWGDTVSDISVKFGVDTRKSLEANGLSA-NSIIFPFTTLLIPL

Ac_LYR3 YLLSYLITPGDWVSRISRKFSVDTGNTLQANEISEDDPTIYPFTTLLVPL

Lu_LYR3 YLLSYVVTWGDFVSSASVRFGGDTGWTLQANGLTEKSATIFPFTTLLVPL

Pp_LYR3 YLLTYIIAQGDYVSKISATFDSDTGRTLEANGLSEQASTIYPFTTLLVPL

Fv_LYR3 YLMSYLITPGDFVSKISDRFDTDTGRTLEANGLSEEAANIYPFTTLLVPL

Ccl_LYR3 YLLSYLVEGDNTVYGISKRFGVDTDRTLEANGLSEGAPNIYPFTTLLVPL

Csi_LYR3 YLLSYLVKGGNTVYGISKRFGVDTDRTLEANGLSEGAPNIYPFTTLLVPL

Pt_LYR3_2 YLLSYLVTPGDDVPAISEQFGAATGRTLEANGLPEQNPTIFPFTTLLIPL

Pe_LYR3_2 YLLSYLVTPGDDVPAISEQFGAATGRTLEANGLPEQNPTIFPFTTLLIPL

Rc_LYR3 YLLSYLVTWGDTVSAVSVKFGGNTGRSLEANGLSEQTPTIYPFTTLLIPL

Pt_LYR3_1 YLLSYLVTWGDTVSIAGVRFGADIGRALEANEISEKNPTIYPFTTLLIPL

Pe_LYR3_1 YLLSYLVTWGDTVSIASVRFGADIGRALEANEISEKNPTIYPFTTLLIPL

Vv_LYR3 YLMSYLVASGDYVSSISVRFGVDTGMTLEANELSEQNPNIYPFTTLLIPL

PanLYK6 YLVSYLVAKGDFVYSIATRFGSDTGWTLVANGLSEENPTIYPFTTLLVPL

Prig_LYK6 YLVSYLVAKGDFVYSIATRFGSDTGWTLVANGLSEENPTIYPFTTLLVPL

Prug_LYK6 YLVSYLVAKGDFVYSIATRFGSDTGWTLVANGLSEENPTIYPFTTLLVPL

Tlev_LYK6 YLVSYLVAKGDFVYSIATRFGSDTGWTLVANGLSEENPTIYPFTTLLVPL

Tori_LYK6 YLVSYLVAKGDFVYSIATRFGSDTGWTLVANGLSEENPTIYPFTTLLVPL

PanLYK8 YLVSYLVGELDSSSSISERFKVDNDLLLSANGLSDPDTTIYPNTTLLVPL

Prig_LYK_8 YLVSYLVGELDSSSSISERFKVDNDLLLSANGLSDPDTTIYPNTTLLVPL

Tlev_LYK8 YLVSYLVGELDSSSSISERFKVDNDLLLSANGLSDPDTTIYPNTTLLVPL

Tori_LYK8 YLVSYLVGELDSSSSISERFKVDNDLLLSANGLSDPDTTIYPNTTLLVPL

Prug_LYK8 YLVSYLVGELDSSSSISERFKVDNGLLLSANGLSDPDTTIYPNTTLLVPL

Mn_LYR3 YLMSYLTDADDDDSKISEAFSVDNELLLKTNLLSDLDNTIYPSTTILVPL

Acom_LYR3 YLMSYLVDPGDTIYIISRMFGVQQQSILDANALSNS-NNIYPFTTLLIPL

Atr_LYR3 YLLSYLVIQGDSISSVSLKFSVSPETILDANELSKENSDIYPLTTLLIPL

Aip_LYR3_1 YLLSYLVDWGDSVSFISQMFNVTTQITLDANSLTMS-SVIYPFTTMLVPL

Adu_LYR3_1 YLLSYLVDWGDSVSFISQMFNVTTQITLDANSLTMS-SFIYPFTTILVPL

Aip_LYR3-2 YLLSYLVSFGDDVSHISERFGVTIETILEANSLSSQKPTINPFTTLLVPL

Adu_LYR3_2 YLLSYLISFGDDVSQISERFGVTIETILEANSLSSQKPTINPFTTLLVPL

Lan_LYR3_2 YLISYLVKPDDDVSQVSERFGVTIETILEANSLSLQQPTINPFTTLLVPL

PvLYR3 YLLSYLVKPDDFVSVIAEKFGVGTMTTLEANSLTLSQSTIYPFTTLLIPL

Lan_LYR3_1 YLLSYLVKGDDSVSLVSEKFGVKTETTLEANNLTMTNATIQPFTTLLVPL

Lj_LYR3 YLLSYLVNWGDSISVISEKFGVSCNNTLEANSLSLTKAKIYPFTTLLVPL

Mt_LYR3 YLLSYLVDWGDSVSFISDKFGVNFRTTLEANTLSLTQSTIYPFTTLLVPL

Ca_LYR3 YLLSYLVNWGDSVSLISDKFGVNFRTTLEANTLSLTQATIYPFTTLLVPL

Cca_LYR3 YLLSYLVKWGDSVSLVSEKFGVNFMTTLEANTLTLTQSMIYPFTTLLVPL

PsLYR3 YLLSFLVNWGDSVSLISEKFGVNFKTTLVANTLTLTQATIYPFTTLLVPL

Gm_LYR3_1 YLLSYLVNWGDSVSFISEKFGVNFMSTLEANTLTLTQAMIYPFTTILVPL

Gm_LYR3_2 YLLSYLVNWGDSVSFISEKFGVNFMTTLEANTLTLTQATIYPFTTILVPL
