# Supplementary material for: Evolutionary History of Plant LysM Receptor Proteins Related to Root Endosymbiosis
Source: Front Plant Sci. 2018 Jul 4;9:923. doi: 10.3389/fpls.2018.00923 (PMC6039847; doi:10.3389/fpls.2018.00923)
Supplement: DATA SHEET S1 — Protein sequences, whole protein, LysM and kinase domain alignments, and tree files. [file Data_Sheet_1.ZIP › Supplementary data/LYSM3-NFP alignment.docx]

Csa_NFP_1 FFITYVWQLTDVVSGVRSIFNVSKDANLEDV—-KGVKL--TN---FV---AGEALFIPL

Set_NFP SLITYVWQQDDNMSEVSKLMNTTVDDIAEA---N—-NVT-SS---FA-SVMGPPMLIPV

Ph_NFP SLITYVWQQGDSMSQVSKLMNATADDIAEA---N—-NVT-SS---FT-SVMGPAMLIPV

Zm_NFP_1 SFITYVWQPGDTLSQVSKLMNATADEIAEA---N—-NVT-SSSVSSA-SAAGLPMLIPV

Sb_NFP SFITYVWQAGDTMSQVSKLMNTTEDEIAEA---N—-NVT-SS-SASA-SLVGQPMLIPV

Zm_NFP_2 SLITYMWQAGDTMSQVSKLMNATVDEIAEA---N—-NVT-ANTSASA-SFVGQPMLIPV

Os_NFP LLVTYVWQPGDDVSVVSALMNASAANIAAS---N—-GVA-GN---ST-FATGQPVLIPV

Bd_NFP YHITYLWRPEDDMSTVSTLMNSSKSDIAEA---N—-NVT-TE---FT-SPTAQPMLIPV

Ta_NFPa LLVTYMWRPVDTMPEVSNLMGSDVGAIAAV---N—-NVS-AD---FT-STTMLPMLVPV

Ta_NFPd LLVTYMWRPVDTMSEVSKLMNSSASAIAAV---N—-NVS-AD---FT-STTMLPMLIPV

Klax_NFP_1 FLITYVLQPGDDLPTLSSLFKTSPDDITSE---N—-NF--TD---FV-TASGLPILIPV

Klax_NFP_2 FLITYVLQPGDDLPTLSSLFKTSPDDITSEN—-N—-NF--TD---FV-TASGSPILIPV

Csa_NFP_2 YFITYIWQPTDNISVVSNEFNVSSDSVLAE---N—-NY--TN---MK-DAANLPVFIPL

Lu_NFP FLITYVWRQGDDLQTVAHKLDASVNDIGLA---N—-NY--MN---FS-EAVNRPVLVPI

Atr_NFP FLITYVWQKGDNIVEVSKRLNTSVERIEDA---N—-KY--RN---FT-AAMDLPVLIPV

Ac_NFP HFLTYVWQTNDDVLSVSSRLNASPVDVRTQ---N—-NY--SN---FS-DAVRLPILIPV

Fv_NFP YLLTYVWQTNDDIFRVSAKFNISALNISGA---N--NF--DN---GS-PVVGQPVLIPL

Pax_NFP YIVTYVWQPGDDVLPVSTMFGASAVDILEA---N—-NY--RN---FT-AAICLPVVIPV

Pin_NFP YIVTYVWQPGDDVLPVSTMFGASAVDILEA---N—-NY--RN---FT-AAICLPVVIAV

Nb_NFP YLVTYVWQPWDDILPVSNMFGASAADILAA---N—-NY--RN---FT-GAICLPVLVPV

St_NFP YLVTYVWQPWDDVLPVSNMFGASAADILAA---N—-NY--RN---FT-AAIYSPVLIPI

Sl_NFP YLVTYVWQPWDDVLPVSNMFGASAADILAA---N—-NY—-RN---FT-AAICSPVLIPV

Egut_NFP YLITYVWQPSDDLLSVSTMFQSEVSDIVTE---N—-NN--RN---FT-ASICLPVFIPV

Si_NFP YLITYIWQPGDQMLSVSSMFNASISDIVME---N-—NN--RN---FS-AAICLPVLIPV

Cp_NFP YFITYVWQPNDDISHVSTKFNASQVHIRAE---N—-RY--EN---FS-DATDFPILVPV

Vv_NFP YLITYVWQPGDDVLLVGTNLKASPVDIRDE---N—-NN--LN---FS-ASVDQPVLIPV

Me_NFP YLITYVWQPDDDVFNVAAKLNASPHDIAIE---N—-NY--RD---FS-VAVHHPLLIPV

Rc_NFP YLITYVWQPEDDIFKVGAKFNASPHDIAIQ---N—-NY--WD---FS-TAVHHPLLIPV

Pt_NFP_1 HLITYVWQPGDDLKKVAAMLNASERNIVIE---N—-NY--DN---FN-AAVYNPIVIPV

Pt_NFP_2 YLITYVWQPSDDLTKVAAKLNASERNIVIE---N—-NY--VN---FT-AAVYLPVLIPV

Md_NFP YHITYIWQPNDDISRVSSRFNVSTLDISSA---N—-NL--HN---DS-AAVELPVVIPV

Pp_NFP HLITYVWQPNDDTFRVSSKFNVSPLDIVTA---N—-DY--RN---FT-AAVGLPVVIPV

Mn_NFP HLITYVWQPTDDIFQVSTKFNTSEVDIINE---N—-NY--RN---FT-DAVGSPLLIPV

Pan_NFP YLITYVWQPSDDIYRVSAMFNASEVDIIIE---N—-NY--QD---FK-AAVGYPVLIPV

Tt_NFP HLITYVWQPIDDIYRVSAMFNASEVDIVTE---N—-NY--RD---FK-AAVGYPVLIPV

Dg_NFP HLISYVWQPMDDVMHVASMFSASSDDIISE---N—-SYY-PN---MS-NATYLPISIPV

Go_NFP YLITYVWKANDNVTLVSSKFGASQGDMLTQ---N-------N---FT-AAANLPILIPV

Mt_NFP YLITYVWQDNDNVTLVSSKFGASQVEMLAE---N--NH---N---FT-ASTNRSVLIPV

Ps_SYM10 HLITYVWQANDNVTRVSSKFGASQVDMFTE---N--N---QN---FT-ASTNVPILIPV

Mt_LYR1 YLITYVWQPNDNLTLVASKLGASPKDIITA---NTNNFG-QN---FT-VAINLPVFIPV

Ca_LYR1 YVITYVWQPNDNVTLVASKFGALPHDIITA---NANNFG-QN---FT-TATNLPVFIPV

Lj_LYS11 YLITYVWHNNDNVSLVASKFGVSTQDIISE---N--NFSHQN---FT-AATNFPILIPV

Aip_NFP YMITYVWQNNDNVSSVAAKFGASAVDILSE---N--NYG-GN---FT-AATYLPVLIPV

Adu_NFP YMITYVWQNNDNVSSVAAKFGASPVDILSE---N--NYG-GN---FT-AATYLPVLIPV

Ca_NFP YLITYVWHANDNVSTVSSKFGASQVDILTE---N--NYN-QN---FA-SAANLPVLIPV

Lan_NFP NLITYVWQPNDTVSTVSSKFGASSADILSE---N--NYG-QN---FT-AAIHQPFLIPV

Lj_NFR5 YLITYVWKPNDNVSLVSAKFGASPADILTE---N--RYG-QD---FT-AATNLPILIPV

Pv_NFP YLITHVWQPNDNVSFVSNKLGASPQDILSE---N—-NYG-QN---FT-AASNLPVLIPV

Gm_NFR5a YLITYVWKPGDNVSLVSDKFGASPEDIMSE---N—-NYG-QN---FT-AANNLPVLIPV

Gm_NFR5b YLITYVWQPSDNVSLVSEKFGASPEDILSE---N—-NYG—QN---FT-AANNLPVLIPV

Ma_NFP FLVTYVWSAEDTVFQLSKKMNSSRDAMEAT---N—-NY—-RN---FS-AAVFHPILIPV

Tc_NFP FFISYVWQPNDSVWSVSAKFNASPLDIVDENKLN—-SY--QN---ISLR-VIPPLMIPV

Gr_NFP YFISYVWQPNDDIWSVSAKFNASAPAIIDENKLN—-DY--QD---ISLA-VIPPLMIPV

Eg_NFP LFITYVWQTNDQVSTVASKFNATPGAIIAE---N—-NQ—-NN---FT-SVIDHPVLIPV

Pan_LYK7 QFITYVWQPNDQVSNVSAKFNTSASEIVNE---N—-KY—-NN---FS-SAVGLPVLIPV

Cg_NFP KLITYVWQQTDDVLRVSETFNASSADIEAE---N—-GY—-QN---YN-DAVGLPVLIPV

Ccl_NFP NLITYVWQPGDDVSQVGAKLNASSAAIETE---N—-GY—-RN---FS-EAVSLPVLIPV

Csi_NFP NLITYVWQPGDDVSQVGAKLNASSAAIETE---N--EY—-RN---FS-EAVSLPVLIPV
